# Tetrahydrohyperforin and Octahydrohyperforin Are Two New Potent Inhibitors of Angiogenesis

**DOI:** 10.1371/journal.pone.0009558

**Published:** 2010-03-09

**Authors:** Beatriz Martínez-Poveda, Luisella Verotta, Ezio Bombardelli, Ana R. Quesada, Miguel Ángel Medina

**Affiliations:** 1 Departamento de Biología Molecular y Bioquímica, Facultad de Ciencias, Universidad de Málaga, Málaga, Spain; 2 Dipartimento di Chimica Organica e Industriale, University of Milan, Milan, Italy; 3 Indena S.p.A., Milan, Italy; 4 Unidad 741 de CIBER “de Enfermedades Raras”, Málaga, Spain; Bauer Research Foundation, United States of America

## Abstract

**Background:**

We have previously shown that hyperforin, a phloroglucinol derivative found in St. John's wort, behaves as a potent anti-angiogenic compound. To identify the reactive group(s) mainly involved in this anti-angiogenic effect, we have investigated the anti-angiogenic properties of a series of stable derivatives obtained by oxidative modification of the natural product. In addition, in the present work we have studied the role of the four carbonyl groups present in hyperforin by investigating the potential of some other chemically stable derivatives.

**Methodology/Principal Findings:**

The experimental procedures included the analysis of the effects of treatment of endothelial cells with these compounds in cell growth, cell viability, cell migration and zymographic assays, as well as the tube formation assay on Matrigel. Our study with hyperforin and eight derivatives shows that the enolized β-dicarbonyl system contained in the structure of hyperforin has a dominant role in its antiangiogenic activity. On the other hand, two of the tested hyperforin derivatives, namely, tetrahydrohyperforin and octahydrohyperforin, behave as potent inhibitors of angiogenesis. Additional characterization of these compounds included a cell specificity study of their effects on cell growth, as well as the *in vivo* Matrigel plug assay.

**Conclusions/Significance:**

These observations could be useful for the rational design and chemical synthesis of more effective hyperforin derivatives as anti-angiogenic drugs. Altogether, the results indicate that octahydrohyperforin is a more specific and slightly more potent antiangiogenic compound than hyperforin.

## Introduction

**Figure 1:**
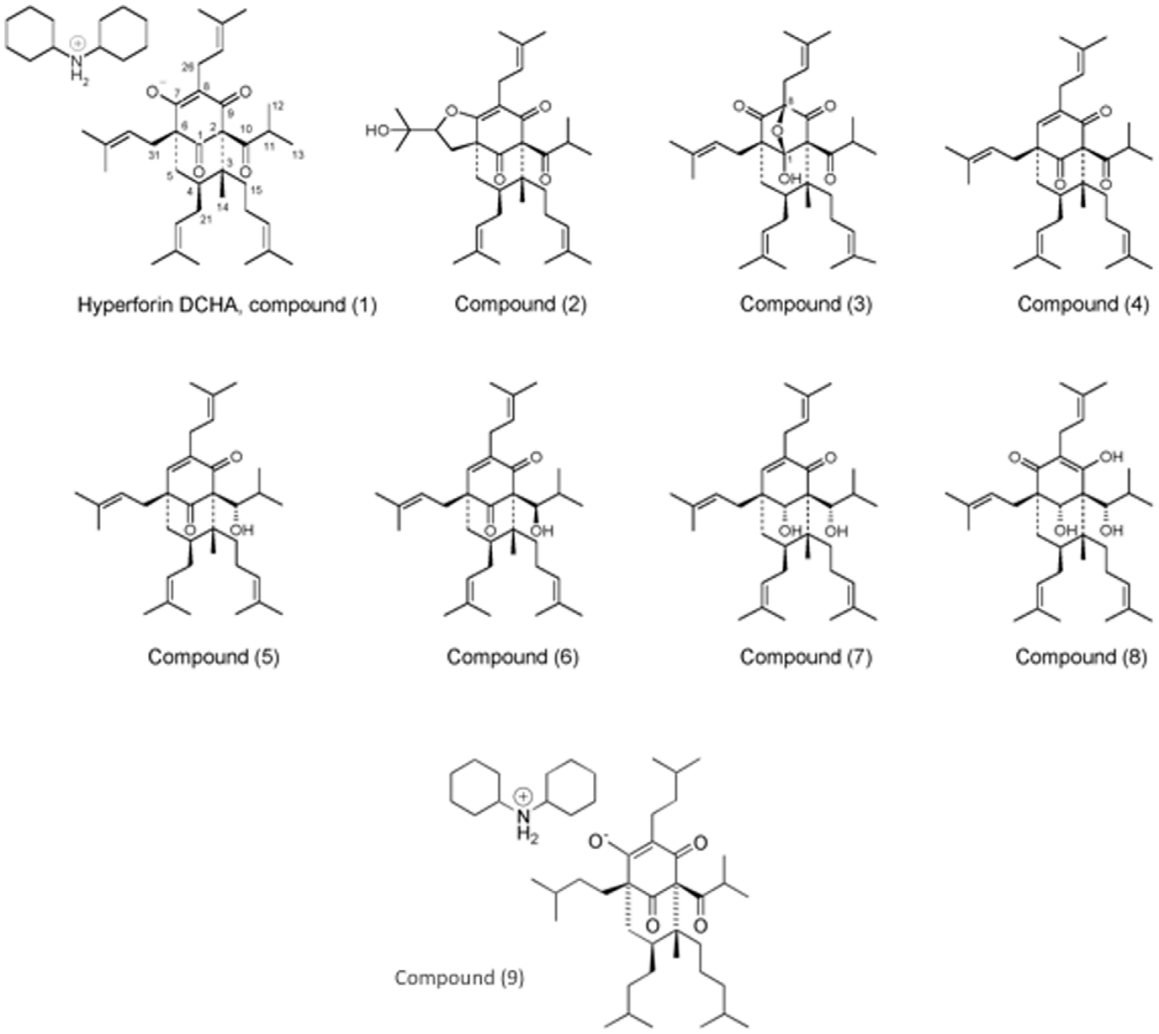
Chemical structure of hyperforin and its derivatives.

St. John's wort (*Hypericum perforatum* L.) is an herbaceous plant that has been known for centuries and used for a variety of medicinal purposes, including the fight against infections and the treatment of respiratory and inflammatory diseases, pectic ulcers and skin wounds [1]. St. John's wort preparations are increasingly popular in the treatment of mild to moderate depression [2], [3]. The main bioactive compound responsible for the antidepressant effects of St. John's wort extracts is its major lipophilic compound, hyperforin (Figure 1, compound **1**). The biomedical relevance of hyperforin is reinforced by the accumulation of scientific evidence pointing to other different effects of hyperforin with potential pharmacological interest. They include effects on Alzheimer disease and as an antibiotic, antiinflammatory, antitumoral and antimetastatic compound [4], [5], [6], [7], [8]. Furthermore, the antiangiogenic potential of hyperforin has been recently unveiled [7], [9], [10], [11].

Angiogenesis, the generation of new blood vessels from the existing vascular bed, has been described as one of the hallmarks of cancer, playing essential roles in tumor growth, invasion and metastasis [12]. In contrast to the highly unstable tumor cells, endothelial cells are genetically stable. On the other hand, tumor blood vessels are different to normal vessels. Therefore, tumor blood vessels are potential targets in therapy for all types of cancer [13], [14]. When resting endothelial cells are activated by an angiogenic signal, they are stimulated to release degrading enzymes allowing endothelial cells to migrate, proliferate and finally differentiate to form new vessels. Any of the steps involved in angiogenesis may be a potential target for pharmacological intervention of angiogenesis-dependent diseases. This is the main reason why angiogenesis has attracted recent attention in the field of pharmacological research [15].

We have previously shown that hyperforin is able to inhibit angiogenesis in an *in vivo* model and behaves as a multi-target antiangiogenic drug by inhibiting several key steps of the angiogenic process. They include inhibition of endothelial cell growth, capillary tube formation on a layer of Matrigel, secretion and production of extracellular matrix degrading enzymes, as well as inhibitory effects on both migrating and invasive potentials of endothelial cells [9]. In another recent work, hyperforin has been shown to block microvessel formation by human dermal microvascular endothelial cells. This research concludes that hyperforin significantly inhibits tumor growth, induces apotosis of tumor cells and reduces tumor vascularisation at concentrations below the toxic effect [10]. It has also been demonstrated that hyperforin restrains polymorphonuclear cell chemotaxis and chemoinvasion and protects against inflammatory events taking place in animal models of angiogenesis [11]. No clear molecular target could, however, be identified. Very recently, hyperforin has been shown to behave also as a potent inhibitor of lymphangiogenesis [16].

Hyperforin (figure 1, compound **1**) is a prenylated phloroglucinol derivative that consists of a phloroglucinol skeleton derivatized with lipophilic isoprene chains. A shortcoming of hyperforin is its chemical and metabolic instability, bound to the presence of reacting functional groups, expressed by the enolized and oxidation –prone β-diketone moiety and the prenyl side chains.

To overcome these issues, we have investigated the anti-angiogenic properties of a series of stable derivatives obtained by oxidative modification of the natural product. Our results throw light on the role of the enolized β-dicarbonyl system contained in the structure of hyperforin and identify two new promising antiangiogenic compounds, one of them even more potent than hyperforin.

## Results

### Effects of Compounds 1–8 on Endothelial Cell Growth

Figure 1 shows the chemical structure of the tested compounds. Octahydrohyperforin (compound **9**) was introduced in the experimental pipeline in a posterior stage of this research, as mentioned below.

Angiogenesis involves local proliferation of endothelial cells. We investigated the ability of hyperforin derivatives to inhibit the growth of bovine aorta endothelial cells (BAEC). Table 1 summarizes these data for the first eight tested compounds, showing that only compound (**8**) had a similar effect to that exhibited by hyperforin (compound **1**), whereas compounds (**2**) and (**3**) had IC_50_ values an order of magnitude higher and compound (**5**) had an IC_50_ value almost two orders of magnitude higher.

**Table 1:** Effects of the tested compounds on the growth of BAEC cells^1^.

Compound	IC_50_ (µM)
1 (hyperforin DCHA)	2.1±0.7
2	12.4±1.5*
3	12.4±3.1*
4	4.5±2.0*
5	84.4±12.7*
6	5.3±1.7*
7	8.0±2.0*
8	1.7±0.1

1 IC_50_ values were calculated from dose-response curves as the concentration of compound yielding a 50% of control cell survival. They are expressed as means±S.D. of three different experiments with quadruplicate samples in each.

*Mean values are significantly higher than that of hyperforin (p<0.05, according to a Student's paired sample test).

### Effects of Compounds 1–8 on Endothelial Cell Migration

**Figure 2:**
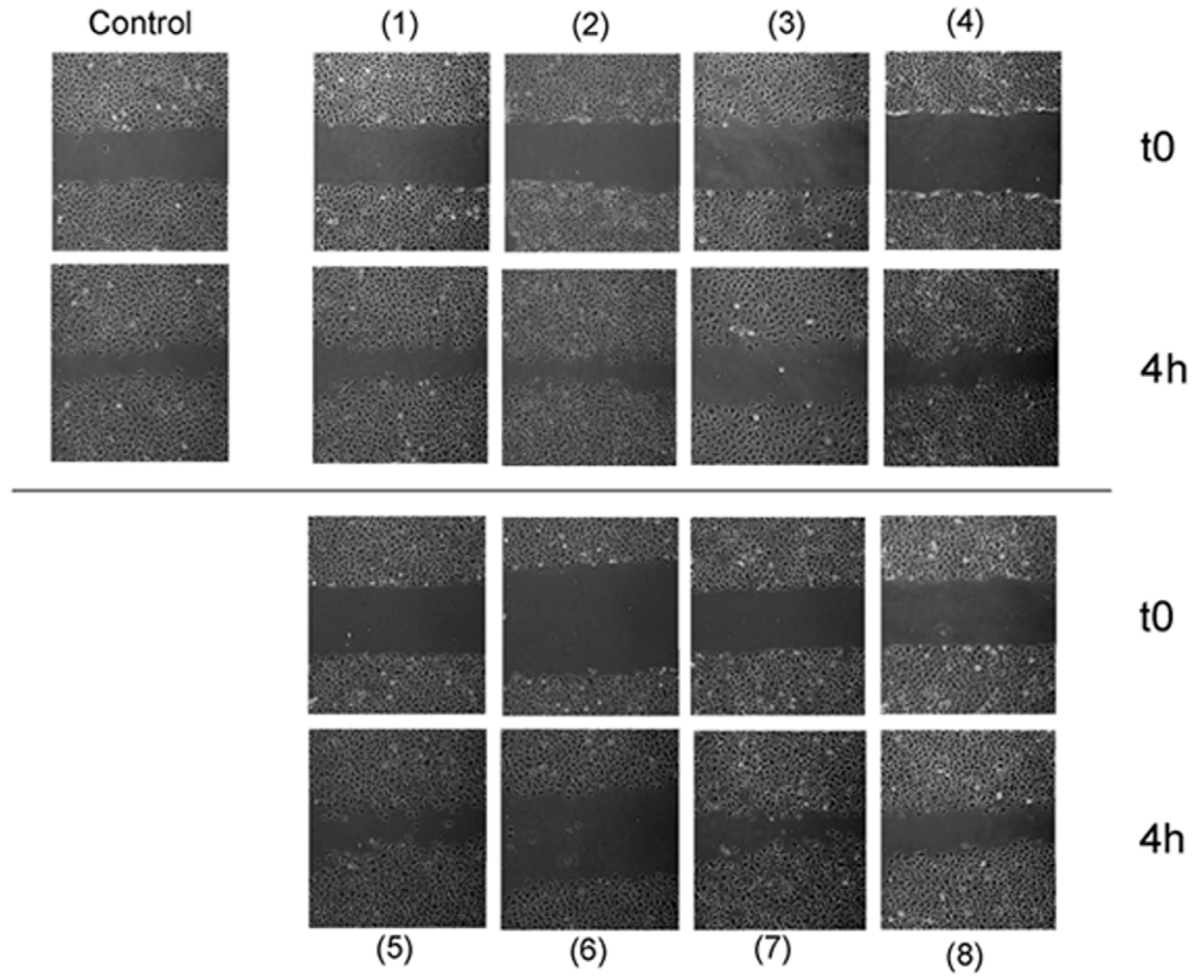
Effects of hyperforin (1) and its derivatives (2–8) in BAEC mobility in a “wound assay”. Confluent monolayers of BAEC were wounded and a wound assay was carried out in the absence or presence of 10 µM of the tested compounds as described in Materials and Methods. Photographs were taken at the beginning of the assay and after 4 h of incubation.

Cell migration is another key step of angiogenesis. The wound assay is frequently used to assess the effects of tested compounds on the migratory potential of adherent cells. As previously described [9], figure 2 shows that hyperforin (compound **1**), at most, only slightly inhibited BAEC migration potential. This seems to be the case for most of the tested hyperforin derivatives, with the exception of compounds (**3**) and (**6**), both showing clear inhibitory effects.

### Effects of Compounds 1–8 on Extracellular Matrix Remodelling Enzymes

**Figure 3:**
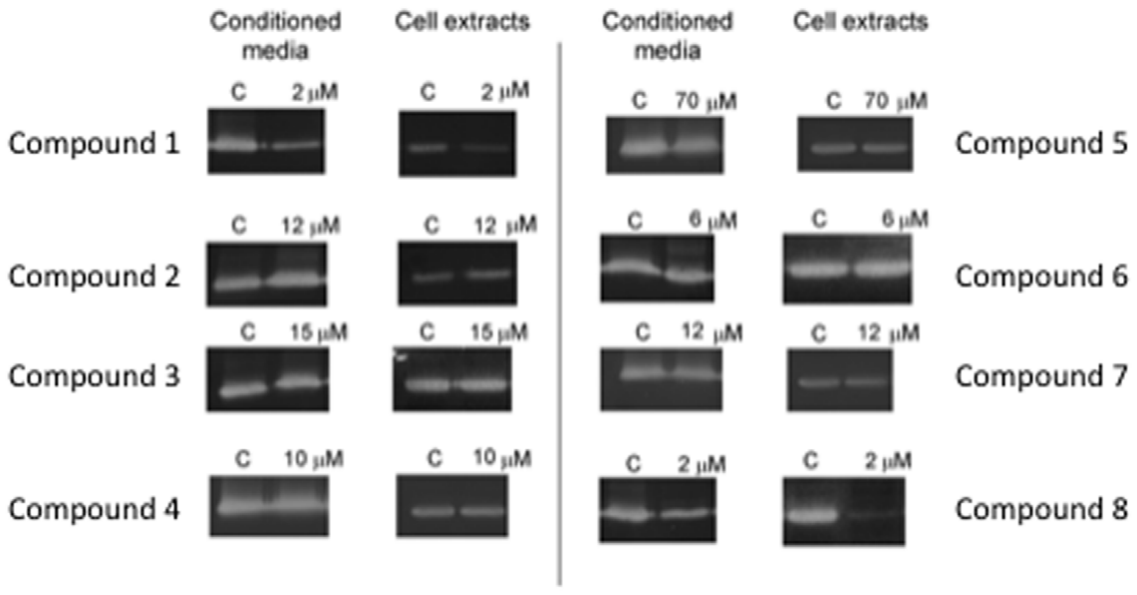
Effects of hyperforin (1) and its derivatives (2–8) on production and secretion of BAEC MMP-2. BAEC cells were treated in the presence of hyperforin derivatives at concentrations in the range of their respective IC_50_ values in the MTT assay for 24 h. Afterwards, conditioned media (for determination of secretion) and cell extracts (for determination of production) were normalized for equal cell density and used for gelatin zymography as indicated in Materials and Methods. Three independent experiments were carried out. Typical results are shown.

Angiogenesis involves the acquisition by endothelial cells of the capability to degrade the basement membrane and to remodel the extracellular matrix. Gelatin zymography of conditioned media and cell extracts of BAEC, untreated and treated for 24 h with hyperforin derivatives at concentrations in the range of their respective IC_50_ values in the MTT assay shows that only hyperforin and compound (**8**) inhibited matrix metalloproteinase-2 (MMP-2) production and secretion (Figure 3). In fact, the reduced compound (**8**) seemed to be a slightly more potent inhibitor of MMP-2 than hyperforin.

**Figure 4:**
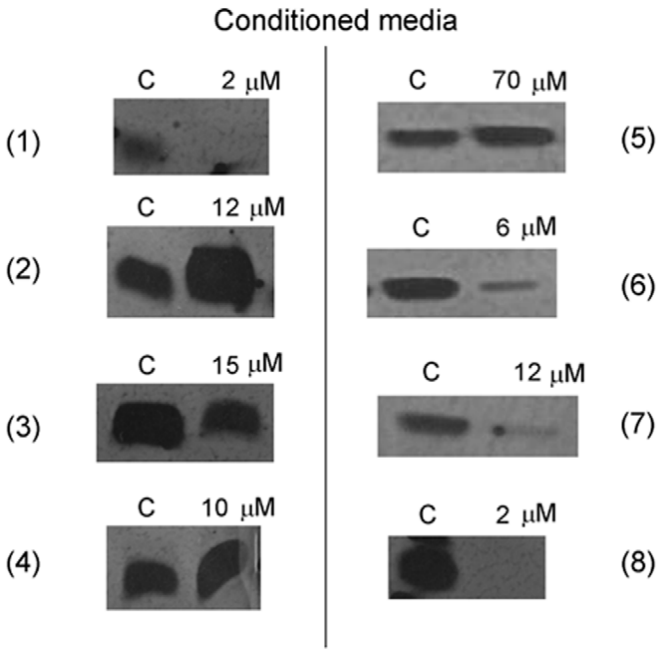
Effects of hyperforin (1) and its derivatives (2–8) on the levels of BAEC urokinase. BAEC cells were treated in the presence of hyperforin derivatives at concentrations in the range of their respective IC_50_ values in the MTT assay for 24 h. Afterwards, conditioned media were normalized for equal cell density and used for the detection of urokinase by plasminogen zymography as indicated in Materials and Methods. Typical results are shown.

Figure 4 shows the expression levels of urokinase-type plasminogen activator (uPA) in conditioned media fromBAEC, untreated or treated for 24 h with hyperforin derivatives at concentrations in the range of their respective IC_50_ values in the MTT assay. Although compounds (**3**), (**6**) and (**7**) showed partial inhibitory effects at higher concentrations, only the reduced compound (**8**) was able to inhibit totally uPA expression at the same concentration at which hyperforin exerted its inhibitory effect. On the other hand, compounds (**2**), (**4**) and (**5**) had no inhibitory effect at all; on the contrary, they seemed to produce an increase in the expression levels of uPA.

### Effects of Compounds 1–8 on Tubule Formation of Endothelial Cells on Matrigel

**Figure 5:**
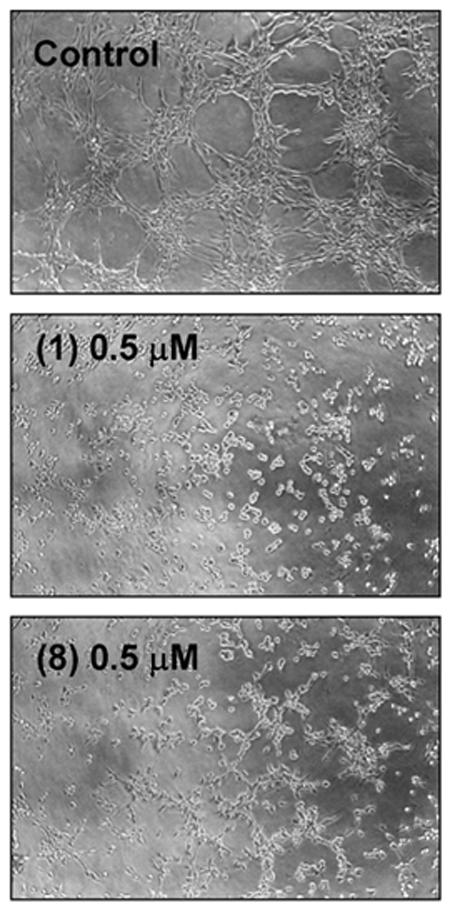
Inhibitory effect of hyperforin (1) and compound (8) on BAEC tubule-like structure formation on Matrigel. Treatments with 0.5 µM hyperforin (1) and compound (8) were carried out as described in Materials and Methods. Cells were photographed 7 h after seeding under an inverted microscope (x40).

The final event during angiogenesis is the organization of endothelial cells in a three-dimensional network of tubes. In vitro, endothelial cells plated on Matrigel align themselves forming tubule-like structures. Table 2 summarizes the effects of the tested compounds on this assay. The minimal inhibitory concentration (MIC) of hyperforin yielding inhibition of endothelial “morphogenesis” on Matrigel was 0.5 µM. Only compound (**8**) had the same MIC value. Figure 5 shows that, in fact, compound (**8**) has a similar inhibitory effect to that exhibited by hyperforin in this assay.

**Table 2:** Effects of the different tested compounds on the assay of tubule-like structures on Matrigel^1^.

Compound	MIC (µM)
1 (hyperforin DCHA)	0.5
2	10.0
3	5.0
4	5.0
5	100.0
6	10.0
7	25.0
8	0.5

1 Minimal inhibitory concentrations (MIC) were those inducing a clear inhibitory effect on the assay of tubule-like structure formation on Matrigel after 7 h of incubation. Each concentration was tested in duplicate, and two different observers evaluated the inhibition of tube formation.

### Second Phase of the Work: Comparison of Hyperforin, Tetrahydrohyperforin and Octahydrohyperforin

Up to this moment, the results obtained altogether showed that only compound (**8**), namely, tetrahydrohyperforin exhibited antiangiogenic effects similar to those shown by hyperforin (compound **1**). To proceed further, we decided to focus our additional experiments on these two compounds and an additional one (compound **9**), tightly related to tetrahydrohyperforin: the satured compound octahydrohyperforin (Figure 1). Firstly, we repeated all the previous experimental setups with this new tested compound. Octahydrohyperforin inhibited the growth of BAEC with an IC_50_ value of 1.0±0.4 µM, which is 50% lower than that obtained with hyperforin. This difference was statistically significant (p<0.05, according to a Student's paired sample test). Effects of octahydrohyperforin on endothelial cell migration and on extracellular matrix remodeling enzymes were similar to those obtained with hyperforin and tetrahydrohyperforin, but at concentrations of octahydrohyperforin that were half of those for these compounds (results not shown). The minimal inhibitory concentration (MIC) of octahydrohyperforin yielding inhibition of endothelial “morphogenesis” on Matrigel was 0.25 µM, that is, also a half of those obtained with hyperforin and tetrahydrohyperforin.

### The Inhibitory Effect of Octahydrohyperforin on Cell Growth Is More Specific for Endothelial Cells than those of Hyperforin and Tetrahydrohyperforin

Table 3 summarizes the results obtained in the MTT assay with the three tested compounds using two non-endothelial cell lines. For both hyperforin and tetrahydrohyperforin, the IC_50_ values were slightly higher than those obtained with endothelial cells. In contrast, IC_50_ values for octahydrohyperforin were 9-fold higher in breast tumor cells and 50-fold higher in fibroblasts than those obtained for this compound in the case of endothelial cells.

**Table 3:** Effects of hyperforin, tetrahydrohyperforin and octahydrohyperforin on the growth of non-endothelial cells^1^.

	MDA-MB231 cells	NIH-3T3 cells
Compound	IC_50_ (µM)	IC_50_ (µM)
1 (hyperforin DCHA)	5±0	15±0
8 (tetrahydrohyperforin)	2±0	13±3
9 (octahydrohyperforin)	9±1*	50±25*

1 IC_50_ values were calculated from dose-response curves as the concentration of compound yielding a 50% of control cell survival. They are expressed as means±S.D. of two different experiments with quadruplicate samples in each.

*Mean values are significantly higher than that of hyperforin (p<0.05, according to a Student's paired sample test).

### *In Vivo* Matrigel Plug Assay of Angiogenesis: Octahydrohyperforin Is a More Potent Inhibitor than Hyperforin

**Figure 6:**
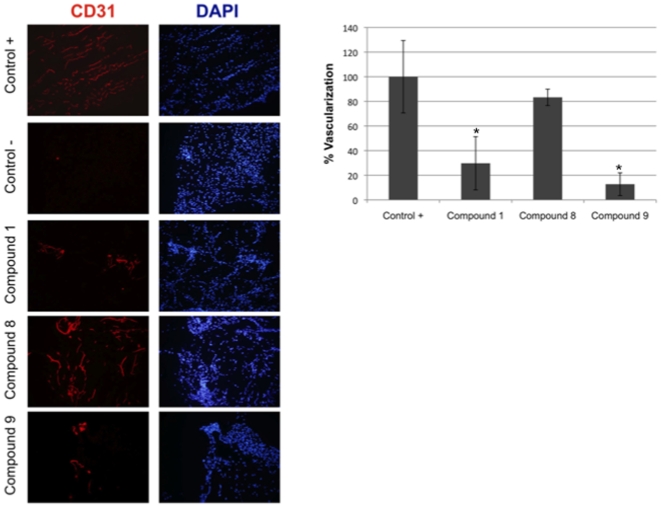
Inhibitory effects of hyperforin (1), tetrahydrohyperforin (8) and octahydrohyperforin (9) on the Matrigel plug assay. *In vivo* experiments with 10 nmol of compound per plug in treatments were carried out as described in Materials and Methods. CD31 positive areas (corresponding to endothelial cells) were quantified using ImageJ software, relativized to total number of DAPI stained nuclei, and all data were expressed as means ± SD of duplicate plugs normalized to the positive control (100% of vascularization). *Mean values are significantly higher than that of positive controls (p<0.05, according to a Student's paired sample test).

Figure 6 shows that, in the *in vivo* Matrigel plug assay of angiogenesis, tetrahydrohyperforin behaved as a less potent inhibitor than hyperforin and octahydrohyperforin. Although the dispersion of experimental data yields non-significant differences between values obtained for these two compounds, the mean values point to a slightly more potent inhibitory effect of octahydrohyperforin.

## Discussion

We have previously shown that hyperforin is a potent multi-target antiangiogenic compound [9]. This observations adds to the antimetastasic effect previously reported and was confirmed by other authors, using stable salts of the bioactive compound [6], [10], [11]. In the present study, we have used dicyclohexylammonium hyperforinate (hyperforin-DCHA, compound (**1**) in Figure 1) as a stable form of hyperforin maintaining its bioactivity. In fact, our results with compound (**1**) as a positive control compound show similar results to those published for the free acid form at slightly lower concentrations, as expected for a stabilized form of the compound [9].

Hyperforin instability is due to the contemporary presence of fastly reacting functional groups: an enolized β-diketone moiety, apparently present in solution as 7-hydroxy, 9-keto tautomer due to the formation of a hydrogen bonding between the ketone in position 1 and the 7-hydroxy group, and the close proximity of this latter to the double bond of the 6- prenyl group. In addition, carbon 8 is strongly nucleophilic, and easily oxidized. Both these characteristics induce a fast reactivity toward oxidizing agents, including light, and lead to unexpected derivatives, some of which also accumulate in the extracts, like compounds (**2**) and (**3**) [17], [18]. One of the major degradation routes for hyperforin is the formation of furan derivatives by mutual oxidative interaction of the enol moiety and the prenyl chains, irreversibly blocking the 7-hydroxy in an ether linkage [19], [20], [21]. In compound (**3**), a hemiacetal species is formed by the introduction of an electrophilic oxygen at C8, which *in situ* reacts with the spatially faced carbonyl group at C1.

Compounds (**2**) and (**3**) were chosen among the different oxidized derivatives to be investigated for their antiangiogenic potential. They represent very stable hyperforin derivatives, where the overall molecular structure is preserved but the enolized β-diketone functionality has collapsed to form furan rings. In previous works, compounds (**2**) and (**3**) have shown to be less active than hyperforin *in vitro* as inhibitors of synaptosomal serotonin reuptake, but they had a comparable effect as growth inhibitors of *P. falciparum* cultures, although showing less toxicity [17], [22]. Oxidized hyperforin derivatives (**2**) and (**3**) have also shown to be equally or more potent than hyperforin as inhibitors of 5-lipooxygenase activity [23]. In addition, furohyperforins are also reported to potently inhibit CYP3A4 enzyme activity [24] thus inferring the enolized β-diketone moiety a significant role in modulating many kinds of activities. Our results herein presented altogether show that these compounds behave as much less potent antiangiogenic compounds than hyperforin. This is evidenced by the limited activity shown in all the panel of tests used.

The role of the four carbonyl groups, functionalities that could be involved in hydrogen bondings with enzymes active sites, was also investigated. The reaction of hyperforin with different reducing agents produced compounds (**4**) to (**8**) [25]. Compounds (**4**) to (**7**) are formally 7-deoxohyperforins, where the C1-C10 non enolizable β-diketone moiety survived to reduction, like in compound (**4**), or was partially reduced to a 10-oxymethine (compounds **5** and **6**) or totally reduced to the 1,10 diol (compound **7**). Interestingly enough, compounds (**4**) to (**7**) have no relevant effects as anti-angiogenic compounds. In these cases the molecules loose a number of intra and inter molecular bondings, while modifying the relative spatial distribution of the oxygenated functions. All of them are much less active than hyperforin, but we should stress that compound (**5**) is the worst, with IC50 and MIC values much higher than the other tested compounds.

The most relevant activities (equal or slightly more potent than those exhibited by hyperforin-DCHA) were observed on compound (**8**), formally a tetrahydrohyperforin, whose enolized β- diketone moiety is reversed with respect to the natural product (9-OH, 7-keto versus 7-OH, 9-keto). This is due to the formation of a strong intramolecular hydrogen bond between the donor 9-OH group and the acceptor hydroxyl at 10 position, which also draws the stereochemical control of the reaction, only producing the 10*S* stereoisomer. Apparently, compound (**8**) is particularly stable if compared to hyperforin and this can be attributed to the strong intramolecular hydrogen bonding that produces orthorombic crystals [25].

Altogether, the results discussed above indicate that only compound (**8**), namely, tetrahydrohyperforin exhibits antiangiogenic effects similar to those shown by hyperforin (compound **1**). To proceed further, we decided to focus our additional experiments on these two compounds and an additional one (compound **9**): the satured compound octahydrohyperforin (Figure 2), obtained by catalytic hydrogenation of hyperforin. This compound is devoid of the rapid oxidative degradation due to the presence of prenyl double bonds in hyperforin, it appears to be a stable derivative and it is endowed of increased lipophilicity. In all the tested *in vitro* assays, octahydrohyperforin behaved as an inhibitor more potent than hyperforin. Furthermore, its stronger antiproliferative effects on BAEC as compared with non-endothelial cells suggest that octahydrohyperforin is more specific for endothelial cells than hyperforin itself. Finally, octahydrohyperforin also behaves as the most potent inhibitor in an *in vivo* Matrigel plug assay of angiogenesis.

In conclusion, we can assert that the enolized β-dicarbonyl system is peculiar for the biological activity of hyperforin as an anti-angiogenic compound, whichever tautomer is present in solution, since the products devoid of this functionality are inactive or less active. Apparently the C1 and C10 carbonyl groups and the prenyl double bonds are not essential to maintain the activity, as shown by the behavior of compounds (**8**) and (**9**). Altogether, our results identify tetrahydrohyperforin and octahydrohyperforin as two new potent inhibitors of angiogenesis and unveil the central role played by the enolized β-dicarbonyl system in the anti-angiogenic effect of hyperforin. On the one hand, these data could be useful for the rational design and chemical synthesis of more effective hyperforin derivatives as anti-angiogenic drugs. On the other hand, the potential of tetrahydrohyperforin and octahydrohyperforin as antiangiogenic compounds deserves to be studied more in depth, including a molecular characterization of their effects on specific targets. Future experimental efforts in both directions seem to be warranted.

## Materials and Methods

### Chemical Compounds

Dicyclohexylammonium hyperforinate (hyperforin-DCHA), a stable form of hyperforin (compound **1**), was gently provided by Indena S.p.A. (Milan, Italy). Compounds **2** to **9** were synthesized as previously described [25]. Stock solutions (10 mg/mL) were prepared in dimethyl sulfoxide (DMSO) and stored in aliquots at −20°C. In all the assays, the vehicle (DMSO) was at less than 1% (v/v) and controls with the vehicle alone were carried out in parallel.

### Cell Culture and Reagents

Cell culture media were purchased from Gibco (Grand Island, NY, USA) and Cambrex (Walkersville, MD, USA). Fetal bovine serum (FBS) was a product of Harlan-Seralab (Belton, U.K.). Matrigel was purchased from Becton Dickinson (Bedford, MA, USA), and Calcein-AM was from Molecular Probes (Eugene, OR, USA). Supplements and other chemicals not listed in this section were obtained from Sigma-Aldrich. Plasticware for cell culture was supplied by NUNC (Roskilde, Denmark). Bovine aortic archs were isolated from calfs immediately after their sacrifice at the local slaughterhouse Famadesa (Málaga), transported to the lab immersed in PBS containing penicillin-streptomycin and amphotericin at standard cell culture concentrations, and used immediately upon arrival for isolation of primary bovine aortic endothelial cells (BAEC) by a collagenase treatment and maintained as previously described [26], [27]. Briefly, BAEC were grown in Dulbecco's modified Eagle's medium (DMEM) containing 1 g/L glucose, 10% FBS, 2 mM glutamine, 50 U/mL penicillin, 50 µg/mL streptomycin, 1.25 µg/mL amphotericin B. Cells were maintained at 37°C and humidified 5% CO_2_ atmosphere. Human MDA.MB231 adenocarcinoma cells and mouse NIH-3T3 fibroblast were maintained as recommended by suppliers (ATCC).

### Cell Growth Assay

The 3-(4,5-dimethylthiazol-2-yl)-2,5-diphenyltetrazolium bromide (MTT; Sigma Chemical Co., St. Louis, MO) dye reduction assay in 96-well microplates was used. The assay is dependent on the reduction of MTT by mitochondrial dehydrogenases of viable cell to a blue formazan product, which can be measured spectrophotometrically. BAE cells -and, in the second phase of this experimental work, also MDA-MB231 and NIH-3T3 cells- (3×10^3^ cells in a total volume of 100 µL of complete medium) were incubated in each well with 1∶1 serial dilutions of compounds to be tested, beginning with 0.1 mM of the compound and down to concentrations in the submicromolar range. After 3 days of incubation (37°C, 5% CO_2_ in a humid atmosphere), 10 µl of MTT (5 mg/ml in PBS) were added to each well and the plate was incubated for a further 4 h (37°C). The resulting formazan was dissolved in 150 µl of 0.04 N HCl/2-propanol and read at 550 nm. All determinations were carried out in quadriplicate. IC_50_ values were calculated as those concentrations of the tested compounds yielding a 50% cell survival.

### Cell Viability Assay

In order to check the viability of endothelial cells after the treatment with hyperforin derivatives in the “tubulogenesis”, migration assay and zymographies, BAE cells were incubated in 96-well plate with the tested compounds in the same conditions used for the aforementioned assays (that means, higher cell densities and shorter incubation times than those employed in the cell growth assay). After the maximum incubation time for these assays (4–24 h), cell viability in comparison to untreated control cells was determined by the addition of MTT as described for cell growth assay.

### Tube Formation by Endothelial Cells on Matrigel

Matrigel (50 µL of about 10.5 mg/mL) at 4°C was used to coat each well of a 96-well plate and allowed to polymerise at 37°C for a minimum of 30 min. 5×10^4^ BAE cells were added with 200 µL of DMEM. Finally, different amounts of hyperforin derivatives were added and incubated at 37°C in a humidified chamber with 5% CO_2_. After 7 h incubation, cultures were observed (40x magnifications) and photographed with a NIKON inverted microscope DIAPHOT-TMD (NIKON Corp., Tokyo, Japan). Each concentration was tested in duplicate, and two different observers evaluated the inhibition of tube formation. Only those assays where no tubular structure could be observed were evaluated as positive in the inhibition of morphogenesis of endothelial cells on Matrigel.

### Endothelial Cell Migration Assay

The migratory activity of BAEC was assessed using a wounded migration assay. Confluent monolayers in 6-well plates were wounded with pipette tips following two perpendicular diameters, giving rise to two acellular 1 mm-wide lanes per well. After washing, cells were supplied with 1.5 mL complete medium in the absence (controls) or presence of 10 µM hyperforin derivatives. Wounded areas were photographed. After additional 4 h of incubation, plates were observed under microscope and photos were taken from the same areas as those recorded at zero time. Acellular surface was determined by image analysis in both controls and treated wells and normalized respect to their respective values at zero time.

### Conditioned Media and Cell Lysates

To prepare conditioned media and cell lysates, BAE cells were grown in 6-well plates. When the cells were at 75% confluency, medium was aspirated, cells were washed twice with phosphate-buffered saline (PBS) and each well received 1.5 mL of DMEM/0.1% BSA containing 200 KIU of aprotinin/mL. Additionally, some wells received hyperforin derivatives at the concentrations mentioned in Results. After 24 h of incubation, conditioned media were collected. The cells were washed twice with PBS and harvested by scrapping into 0.5 mL of 0.2% Triton X-100 in 0.1 M Tris/HCl containing 200 KIU of Trasylol/mL. Media and cell lysates were centrifuged at 1000x*g* and 4°C for 20 min. Afterwards, the supernatants were collected and used for zymography. Duplicates were used to determine cell number with a Coulter counter.

### Zymographies

Assays of urokinase-type plasminogen activator (uPA) activity in gel were carried out as follows. Aliquots of cell lysates normalized for equal cell numbers were subjected to sodium dodecylsulfate-polyacrylamide gel electrophoresis (SDS-PAGE) at 4°C under non-reducing conditions, with 5% stacking gel and 10% resolving gel. Gels were washed for 10 min twice with 50 mM Tris/HCl, pH 7.4, supplemented with 2% Triton X-100 and twice with 50 mM Tris/HCl, pH 7.4 and laid over a substrate gel prepared with agar (0.8%), plasminogen (40 µg/mL) and skimmed milk (1.5% in PBS). Gels were incubated under a moist atmosphere overnight at 4°C and then incubated at 37°C. After 4–8 h, bands of proteolysis due to uPA activity were photographed under dark field.

The gelatinolytic activity of matrix metalloproteinase-2 (MMP-2) delivered to the conditioned media or present in cell lysates was detected in gelatinograms. Aliquots of conditioned media and cell lysates normalized for equal cell numbers were subjected to non-reducing SDS/PAGE as above but with gelatin (1 mg/mL) added to the 10% resolving gel. After electrophoresis, gels were washed twice with 50 mM Tris/HCl, pH 7.4, supplemented with 2% Triton X-100, and twice with 50 mM Tris/HCl, pH 7.4. Each wash with continuous shaking lasted 10 min. After the washes, the gels were incubated overnight at 37°C and immersed in a substrate buffer (50 mM Tris/HCl, pH 7.4, supplemented with 1% Triton X-100, 5 mM CaCl_2_, and 0.02% Na_3_N). In some experiments, hyperforin derivatives at the concentrations mentioned in results were added to the substrate buffer. Finally, the gels were stained with Commassie blue R-250 and the bands of gelatinase activity could be detected as non-stained bands in a dark, stained background.

### *In Vivo* Mouse Matrigel Plug Assay

C57BL/6 female mice were injected s.c. near the abdominal midline, via a 23-gauge needle with 300 mL of Matrigel (Beckton-Dickinson) containing basic fibroblast growth factor (bFGF; 0.5 µg/mL) and 10 nmol of the corresponding compound. Positive control mice received the same volume of Matrigel with bFGF mixed with the same amount of vehicle (DMSO). Negative control mice were injected with Matrigel containing the corresponding dose of PBS and DMSO. After injection, the Matrigel rapidly formed a single, solid gel plug. After 8 days, mice were sacrificed and plugs were removed. Plugs were processed for cryoprotection with increased concentrations of sucrose, embedded in OCT and frozen in liquid nitrogen. Sections of 10 mm thickness were collected on poly-L-lysinated slides and fixed in three stepts of acetone, acetone-chloroform (1∶1) and acetone, keeping the samples at −20° while fixing. Immunodetection of CD31 and DAPI staining were performed and random fields of the sections were photographed under fluorescence microscope. CD31 positive areas were quantified using ImageJ software and all data was expressed as means ± SD of duplicate plugs normalized to the positive control (100% vascularization).

### Statistical Analysis

Statistical significance was determined by the Student's paired sample test. Values of p <0.05 were considered to be significant.
